# Development and validation of a prognostic model incorporating patient reported outcomes for advanced gastric and esophageal carcinoma (AGOC) using individual patient data from two AGITG randomized clinical trials

**DOI:** 10.1007/s10120-025-01654-2

**Published:** 2025-09-16

**Authors:** Sayeda Kamrun Naher, David Espinoza, Peter Grimison, Kohei Shitara, Nick Pavlakis, David Goldstein, Martin R. Stockler, Rebecca Mercieca-Bebber, Katrin Marie Sjoquist

**Affiliations:** 1 NHMRC Clinical Trials Centre, the University of Sydney, Sydney, NSW Australia; 2 Chris O’Brien Lifehouse, Camperdown, NSW Australia; 3 Gastrointestinal Oncology, Department of National Cancer Centre Hospital East, Chiba, Japan; 4 Royal North Shore Hospital, St Leonards, NSW Australia; 5 Prince of Wales Hospital, University of New South Wales, Sydney, Australia

**Keywords:** Gastric cancer, Prognostic model, Patient reported outcomes, Validation

## Abstract

**Background:**

We developed and validated a prognostic model incorporating readily accessible clinicopathological data and specific patient-reported outcomes (PROs).

**Methods:**

We used data from two randomized trials comparing regorafenib to placebo: AGITG INTEGRATE IIa (*n* = 251) for model development and AGITG INTEGRATE (*n* = 152) for validation. Candidate variables were chosen from a systematic literature review and expert consultation. Significant prognostic factors in the multivariable model were identified using univariable Cox proportional hazards models with a *p*-value of < 0.1. Multivariable Cox proportional hazards models were developed using clinicopathological and PRO variables, with model selection refined using least absolute shrinkage and selection operator (LASSO). The model’s discrimination and calibration were assessed using concordance indices (C-statistics) and calibration plots.

**Results:**

Univariable analysis identified 9 clinicopathological variables and 4 PRO domains that were prognostic for overall survival: body mass index (BMI), ECOG performance status, number of metastatic sites, liver involvement, treatment with regorafenib, neutrophil–lymphocyte ratio (NLR), LDH, albumin, CA 19–9, appetite loss, constipation, fatigue, and pain. The initial multivariable model (M1) incorporated geographic region (Asia vs non-Asia), performance status, number of metastatic sites, treatment with regorafenib, NLR, BMI, LDH, CA 19–9, and albumin. The preferred multivariable model (M2), including the abovementioned variables plus the 4 PROs, demonstrated superior discriminative ability with higher C-statistic values than models without PROs. Plots supported the model’s calibration.

**Conclusions:**

Incorporating PROs into prognostic models for AGOC improved the accuracy of survival predictions. Further research is needed to validate its use in routine clinical practice.

## Introduction

Advanced (metastatic or inoperable or unresectable) gastric and esophageal carcinoma (AGOC) is common and has a poor prognosis with the fifth-highest incidence and fourth-highest mortality rates globally [1]. Predicting future survival time is an important and difficult problem for doctors, patients, and carers. The range and complexity of factors influencing prognosis contribute to this difficulty [2]. Patients with advanced cancer who discussed prognosis and life expectancy with their oncologists developed a better understanding of the nature of their illness [3].

Nomograms for estimating prognosis in AGOC have been developed using clinicopathological variables including performance status, presence of liver metastasis, peritoneal metastasis, and neutrophil to lymphocyte ratio (NLR) [4–7]. The majority of previous models were developed in the setting of first-line chemotherapy [4, 5]. Other studies assessed a limited number of variables [6, 7]. The applicability of these models to patients having second and subsequent line systemic therapy is unclear.

Patient-reported outcomes (PROs) collected at baseline have shown prognostic value in other types of advanced cancer [8, 9]. A systematic review by Mierzynska et al. reported on the prognostic value of various baseline PRO scores in 41 studies across 15 cancer types [9]. The domains most frequently associated with survival included physical functioning (17 studies) and global health status and quality of life (15 studies) [9]. Among these studies, Park 2008 reported that patient-reported social functioning, assessed at baseline with the QLQ-C30, was prognostic in advanced gastric cancer in a model including bone metastases, hemoglobin, and age [10]. Our systematic review identified multiple domains of PRO that were prognostic in gastric cancer [11]. Despite broad, growing evidence about the value of PROs in predicting survival, there has been limited development and validation of prognostic models for AGOC that incorporate PROs. [12, 13].

The AGITG INTEGRATE and INTEGRATE IIa are randomized trials of regorafenib in AGOC [14, 15]. These trials showed that regorafenib, a multi-targeted, oral tyrosine kinase inhibitor (TKI), improved progression-free survival and overall survival in AGOC after previous systemic therapies had failed or were poorly tolerated. Both trials included participants with ECOG performance status 0–1 previously treated with at least two lines of systemic therapy.

We sought to improve prognostication by developing and validating a model and nomogram using readily available clinicopathological variables plus a focused set of PROs, using individual patient data from these two clinical trials.

## Methods

We used INTEGRATE IIa (*n* = 251, NCT02773524) for model development and INTEGRATE (*n* = 152, ANZCTR 12612000239864) for validation. Both were randomized controlled trials in similar participants and clinical settings.

### Model and nomogram development - candidate variables

We identified potential prognostic variables by reviewing the literature and consulting with oncologists, quality of life (QOL) experts, statisticians, and members of INTEGRATE Trial Management Committees [11].

Variables tested in univariable analyses included geographical region, Eastern Cooperative Oncology Group (ECOG) performance status, prior therapy lines, neutrophil to lymphocyte ratio (NLR), serum albumin concentration, body mass index(BMI), primary disease site, number of metastatic sites, tumor location; serum concentrations of carbohydrate antigen (CA) 19–9 and lactate dehydrogenase (LDH); previous resection of primary (gastrectomy), age, sex at birth, treatment with regorafenib versus (vs) placebo and the following PRO assessed at baseline with the EORTC QLQ-C30 and QLQ-STO22 (appetite loss, pain, fatigue, lack of mobility, lack of energy, leg swelling, constipation) (See Appendix A).

Prognostic factors included in the multivariable model were identified through univariable Cox proportional hazards models, using a *p*-value threshold of < 0.1. Multivariable models were developed using the least absolute shrinkage and selection operator (LASSO). A 10% missing value threshold was set; variables exceeding this were planned for imputation to prevent sample size reduction. However, as no variable had missing data exceeding the 10% threshold, imputation was not necessary. Standardization was applied to albumin, LDH, and CA 19–9 to ensure comparability.

Cox proportional hazards regression was used for all time-to-event analyses. The proportional hazards assumption was assessed, and this showed strong evidence that the variable for gastrectomy violated this assumption (*p* < 0.001). We therefore stratified all multivariable analyses by gastrectomy (yes versus no).

Geographical region (Asia vs. non-Asia: Australia, USA, Canada, NZ) was included in the multivariable models because it was prognostic in a previous analysis of the INTEGRATE trial, even though it was not prognostic in our current univariable analysis [18].

Three models were developed from the multivariable analyses, all stratified by gastrectomy status. The first model (M1) included only clinicopathological variables. The second model (M2) expanded on M1 by adding PROs from EORTC QLQ-C30. The third model (M3) expanded M2 by including selected variables from the QLQ-STO22. A nomogram was developed for M2, including the variables selected by the LASSO.

The rationale for selecting Model M2 was that the QLQ-C30 is a widely used and readily available generic patient-reported outcome measure (PROM), which includes identified, important PRO domains, with minimal respondent and administrator burden. The rationale for testing Model M3 was its inclusion of a subscale from the QLQ-STO22 specifically assessing pain ‘in the stomach area’ and pain associated ‘with eating’, rather than the generic pain subscale from the QLQ-C30.

### Validation of the model

Internal validation of the models was assessed in terms of discrimination and calibration. Discrimination was assessed with the concordance index (C-index), which assesses the model’s ability to distinguish between individuals who did and did not experience the event of interest (death) [19, 20]. Confidence intervals for the C-index were calculated using bootstrap-resampling from the construction data set (1000 bootstrap samples per model).

External validation was assessed using data from the INTEGRATE trial; this data was not used for model development. Calibration assesses the accuracy of the model’s predicted probabilities of outcomes by comparing them with the actual observed outcomes [21]. Calibration was assessed visually using plots of observed versus predicted probabilities of overall survival at 6 months. The effects of model recalibration were assessed using re-estimation of intercept and re-estimation of both the intercept and the slope.

Analyses were performed using SAS version 9.4 and R version 4.3.2. [16, 17].

## Results

### Descriptive statistics

The baseline characteristics of the 403 participants in INTEGRATE and INTEGRATE IIa are summarized in Table 1. Approximately half the participants were recruited in Asia. The median age was 63 years, 77% were male, and the primary site was stomach in 69%. The median overall survival time was 6 months, with a range from 2 to 30 months.

**Table 1:** Baseline characteristics of the participants

Characteristics	Integrate N (%)	Integrate IIa N (%)	Both groups N (%)
Region			
Asia	54 (36%)	157 (63%)	211 (52%)
Rest of world	98 (65%)	94 (38%)	192 (48%)
Age -Median (IQR)	62(54–68)	64 (57–71)	63 (56–70)
Sex—Male	120 (79%)	190 (76%)	310 (77%)
Primary site			
GOJ	58 (38%)	69 (28%)	127 (32%)
Stomach	94 (62%)	182 (73%)	276 (69%)
ECOG			
0	62 (41%)	93 (37%)	155 (39%)
1	90 (59%)	158 (63%)	248 (62%)
Prognosis (months)- Median (range)	6 (3–18)	4 (2–30)	6 (2–30)
BMI—Median (IQR)	23 (21–27)	22 (19–25)	22(20–26)
Prior lines of treatment			
1–2	152 (100%)	149 (59%)	301 (75%)
3 +		101 (40%)	101 (25%)
Number of metastatic Sites 4 + Liver metastasis	35 (23%) 75 (49%)	52 (21%) 128 (51%)	87 (22%) 203 (50%)
Peritoneal metastasis	47 (31%)	74 (30%)	121 (30%)
Gastrectomy	52 (34%)	99 (39%)	151 (38%)
NLR- Median (IQR)	3 (2–6)	3 (2–5)	3 (2–5)
LDH- Median (IQR)	241 (187–396)	231 (177–355)	234 (178–374)
Albumin—Median (IQR)	37 (35–43)	37 (32–40)	37 (33–41)
Ca 19–9- Median (IQR)	53 (12–309)	53 (13–544)	53 (13–467)
PRO domain score, 0–100 scale (EORTC QLQ-C30)			
Appetite loss			
30 +	91 (60%)	157 (63%)	248 (62%)
< 30	45 (30%)	83 (33%)	128 (32%)
Constipation			
30 +	62 (41%)	107 (43%)	169 (42%)
< 30	74 (49%)	132 (53%)	206 (51%)
Fatigue			
30 +	97 (64%)	170 (68%)	267 (66%)
< 30	39 (26%)	70 (28%)	109 (27%)
Pain			
30 +	61 (40%)	123 (49%)	184 (46%)
< 30	75 (49%)	117 (47%)	192 (48%)
Pain (STO22)			
30 +	54 (36%)	106 (42%)	160 (40%)
< 30	82 (54%)	133 (53%)	215 (53%)

*IQR* interquartile range, *GOJ* gastro-esophageal junction, *ECOG* eastern cooperative oncology group, *BMI* body mass index, *NLR* neutrophil to lymphocyte ratio, *LDH* lactate dehydrogenase, *CA* carbohydrate antigen, *PRO* patient reported outcome, *EORTC QLQ* European Organisation for Research and Treatment of Cancer quality of life questionnaire. The possible scores for these scales ranges from 0 (none at all) to a 100 (worst possible).

Baseline PROs indicated a significant symptom burden, with appetite loss reported in 61%, constipation in 42%, fatigue in 66%, and pain in 46%.

### Univariable and multivariable model

In the development dataset, 9 of 15 clinicopathological variables collected at baseline met the pre-specified threshold in univariable analysis (*p* ≤ 0.1) for potential inclusion in the multivariable model. These factors included ECOG performance status (1 vs 0) (*p* = 0.02), BMI (*p* = 0.08), number of metastatic sites (*p* < 0.001), liver involvement (*p* = 0.04), treatment with regorafenib vs placebo (*p* = 0.005), NLR (*p* < 0.001), LDH (*p* < 0.001), albumin (*p* < 0.001), and CA 19–9 (*p* < 0.001). Univariable and multivariable analyses are summarized in Table 2.

**Table 2:** Univariable and multivariable analyses of associations between baseline characteristics and overall survival time

Variable	Class	Univariable		M1—Multivariable		M2—Multivariable		M3—Multivariable	
		Hazard ratio (95% CI)	*p*-value	Hazard ratio (95% CI)	*p*-value	Hazard ratio (95% CI)	*p*-value	Hazard ratio (95% CI)	*p*-value
Region	Rest of world vs Asia	1.17 (0.89–1.53)	0.26	1.58 (1.15–2.18)	0.005	1.68 (1.20–2.35)	0.0026	1.59 (1.14–2.22)	0.006
Age	10-year increase	0.92 (0.82–1.03)	0.26						
Sex		0.89 (0.66–1.21)	0.47						
Primary Site	Stomach vs GOJ	0.86 (0.64–1.15)	0.32						
ECOG performance status	1 vs 0	1.38 (1.05–1.80)	0.02	1.16 (0.87–1.54)	0.31	1.04 (0.76–1.40)	0.82	1.06 (0.78–1.42)	0.72
BMI	10-unit increase	0.75 (0.55–1.03)	0.08	0.53 (0.38–0.75)	0.0004	0.57 (0.39–0.84)	0.004	0.59 (0.41–0.85)	0.005
Prior lines of treatment	2-unit increase	1.09 (0.88–1.35)	0.42						
Number of metastatic sites	4 + vs 1	2.19 (1.44–3.33)	< 0.001	2.61 (1.66–4.12)	< 0.0001	2.20 (1.39–3.48)	0.001	2.07 (1.29–3.31)	0.001
Liver metastasis	Yes vs no	0.76 (0.59–0.99)	0.04						
Peritoneal Metastasis	Yes vs no	1.19 (0.89–1.58)	0.24						
Treatment	Rego vs Placebo	0.67 (0.51–0.89)	0.005	0.73 (0.54–0.97)	0.03	0.77 (0.57–1.04)	0.09	0.75 (0.55–1.00)	0.05
NLR	6-unit increase	1.26 (1.11–1.44)	< 0.001	1.26 (1.07–1.47)	0.004	1.21 (1.02–1.43)	0.03	1.25 (1.05–1.48)	0.011
LDH	250-unit increase	1.42 (1.20–1.68)	< 0.001	1.40 (1.17–1.67)	< 0.001	1.51 (1.26–1.82)	< 0.0001	1.49 (1.24–1.78)	< 0.0001
Albumin	10-unit increase	0.68 (0.56–0.83)	< 0.001	0.73 (0.60–0.89)	0.002	0.80 (0.65–1.00)	0.05	0.80 (0.64–0.99)	0.04
CA 19–9	200-unit increase	1.01 (1.00–1.01)	< 0.001	1.01 (1–1.01)	< 0.0001	1.01 (1–1.01)	0.0006	1.01 (1–1.01)	0.0003
Appetite loss	33-unit increase	1.43 (1.25–1.64)	< 0.001			1.21 (1.01–1.45)	0.03	1.22 (1.02–1.46)	0.03
Constipation	33-unit increase	1.31 (1.13–1.53)	< 0.001			1.07 (0.89–1.27)	0.48		
Fatigue	33-unit increase	1.50 (1.26–1.80)	< 0.001			1.07 (0.81–1.42)	0.63	1.08 (0.84–1.40)	0.53
Pain	33-unit increase	1.43 (1.24–1.64)	< 0.001			1.18 (0.97–1.44)	0.10		
Pain (STO22)	33-unit increase	1.70 (1.37–2.10)	< 0.001					1.38 (1.07–1.79)	0.013

Baseline PROs that met the univariable analysis threshold for inclusion in the multivariable model included appetite loss (*p* < 0.001), constipation (*p* < 0.001), fatigue (*p* < 0.001), and pain (*p* < 0.001) from the QLQ-C30, and stomach pain (*p* < 0.001) from the QLQ-STO22.

Three multivariable models were constructed from the multivariable analysis, all stratified by gastrectomy status. The first model (M1) included clinicopathological factors such as region, ECOG status, number of metastatic sites (extent of cancer), BMI, treatment, NLR, LDH, albumin, and CA 19–9. The second model (M2) expanded on M1 by adding PROs from EORTC QLQ-C30, specifically fatigue, pain, appetite loss, and constipation. The third model (M3) used the item on stomach pain from the QLQ-STO22, along with the subscales for fatigue and appetite loss from the QLQ-C30, to model M1. Liver involvement was excluded from multivariable models based on the LASSO.

These multivariable analyses (M2) demonstrated several clinicopathological and PRO factors that were prognostic for overall survival in models accounting for the effects of other variables. Survival times were shorter for participants from the rest of the world than from Asia (HR 1.68, 95% CI 1.20–2.35, *p* = 0.0026). Shorter survival times were also associated with number of metastatic sites (HR = 2.20, 95% CI 1.39–3.48, *p* = 0.001), higher NLR (HR = 1.21, 95% CI 1.02–1.43, *p* = 0.03), higher LDH (HR = 1.51, 95% CI 1.26–1.82, *p* < 0.0001), and higher CA 19–9 (HR = 1.01, 95% CI 1–1.01, *p* = 0.0006). Longer survival times were associated with higher BMI (HR 0.57, 95% CI 0.39–0.84, *p* = 0.004), and higher serum albumin (HR = 0.80, 95% CI 0.65–1.00, *p* = 0.05). Performance status, liver metastasis, and lines of previous therapy were not significant in multivariable models.

Among the PRO, appetite loss was associated with shorter survival in both univariable and multivariable analysis (HR = 1.21, 95% CI 1.01–1.45, *p* = 0.03), whereas constipation, fatigue, and pain were significant in univariable models (*p* < 0.001), but not in the multivariable model (*p* > 0.10). The subscale for pain from the QLQ-STO22 had slightly increased prognostic significance than the pain scale from the QLQ-C30.

Overall, factors such as region, number of metastatic sites, treatment, NLR, LDH, albumin, and appetite loss consistently showed significant associations with survival, while other factors like age, sex, and primary site show little to no impact.

### Model performance

The performance of these multivariable models was assessed using the C-statistic (see Table 3). M2 and M3 exhibited higher C-statistics than M1, indicating improved prediction by including PROs. M2, which incorporated the PRO indicators from EORTC QLQ-C30, but not the QLQ-STO22, was chosen as the final model on the basis of its higher C-statistics in both the gastrectomy and non-gastrectomy strata and the benefit of using PRO domains from one, rather than two, separate PRO measures.

**Table 3:** Model’s *C*-Statistic + Bootstrap 95% CIs

Model	Gastrectomy	*C* -statistic	95% CI	
M1	No	0.695	0.660	0.732
	Yes	0.664	0.619	0.714
M2	No	0.723	0.686	0.759
	Yes	0.692	0.645	0.750
M3	No	0.721	0.685	0.756
	Yes	0.685	0.633	0.743

**Fig. 1:**
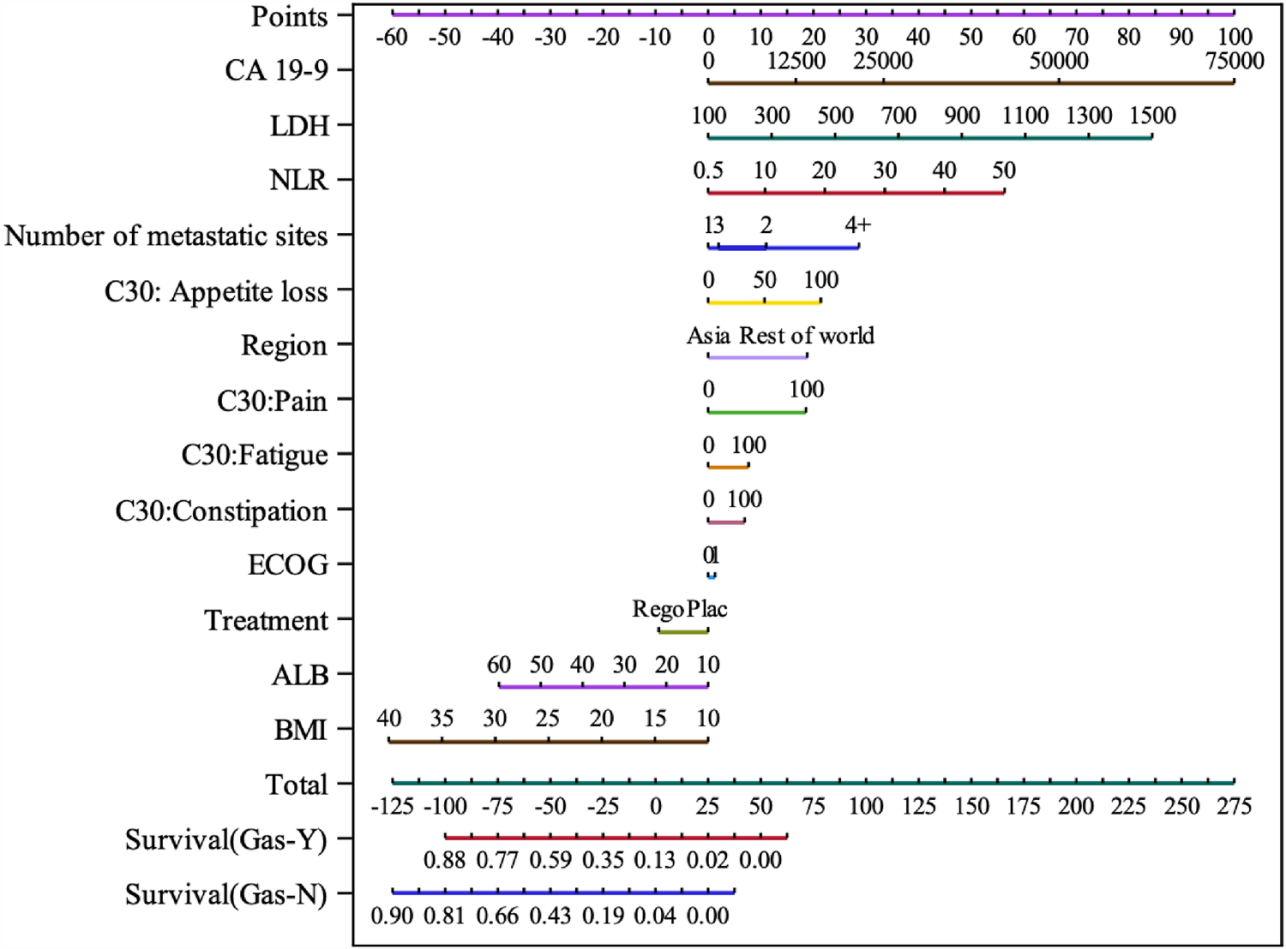
Nomogram for predicted probability of survival at 6 months. *CA* carbohydrate antigen, *LDH* lactate dehydrogenase, *NLR* neutrophil to lymphocyte ratio, *C30* EORTC QLQ-C30, *ECOG* eastern cooperative oncology group, *Rego* regorafenib, Plac placebo, *ALB* albumin, *BMI* body mass index, *Gas* gastrectomy, *Y* yes, *N* no. *The more points the lower the estimated probability of survival at 6 months

The nomogram (Fig. 1) based on Model-2 illustrates the extent to which the various predictor variables affect the probability of overall survival at 6 months. For example, in this dataset, the 4 or more metastatic sites were associated with a much lower probability of survival at 6 months than 3 or fewer metastatic sites, whereas having an ECOG performance status 0 versus 1 had relatively little effect. Appetite loss and pain had larger effects on the probability of survival than fatigue and constipation.

### Validation of the model

#### Calibration

**Fig. 2:**
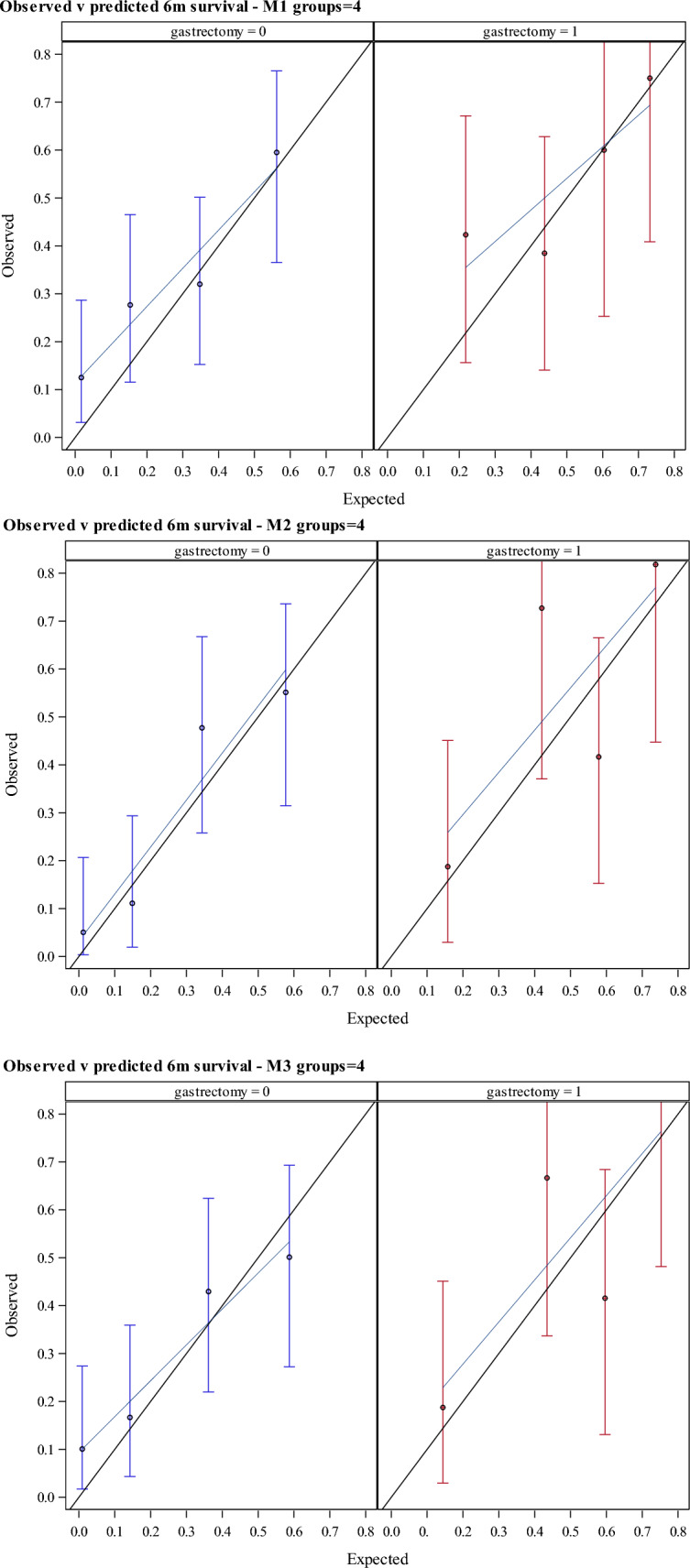
Calibration plot for observed vs predicted 6 months survival in INTEGRATE I trial population

The model’s predictions of 6-month survival were well-calibrated for participants with and without prior gastrectomy, as indicated by the calibration curves being close to the 45-degree reference line reflecting perfect calibration (Fig. 2). Panel B, showing the calibration of M2, indicates the improvement resulting from the inclusion of the PRO for fatigue, pain, and constipation to M1 shown in Panel A. Use of the QLQ-STO22 items for stomach pain in M3 did not improve calibration compared with the use of the generic items for pain in M2 from the QLQ-C30. There was a tendency for the lowest predicted survival probabilities to underestimate the observed survival probability in all the models.

## Discussion

This study advances prognostic modeling for overall survival in AGOC patients by incorporating a broader set of clinicopathological variables and PROs from the EORTC QLQ-C30 and QLQ-STO22, enhancing both accuracy and clinical applicability. Our model M2 incorporates additional clinicopathological variables and patient-reported outcomes including fatigue, alongside known predictors of survival such as pain, appetite loss, and constipation [18]. We selected candidate PROs a priori, based on prior literature and expert consultation, in addition to robust univariable testing ahead of including variables in our multivariable analyses.

Our methodological approach builds on previous work by using a more comprehensive and robust multivariable modeling strategy, with our models that incorporated PROs demonstrating superior predictive performance, reflected by improved C-statistics from 0.695 in M1 to 0.723 in M2 with the inclusion of patient-reported pain, appetite loss, and constipation. The incorporation of these additional variables provided more individualized and accurate estimates of survival probability at 6 months.

Sensitivity analyses demonstrated good calibration with only minor variations observed across different scenarios, and support the use of model M2.

A prognostic model that included baseline clinicopathological variables and selected PRO from the QLQ-C30 provided clinically useful estimates of overall survival. These variables can feasibly be collected in routine clinical practice, using a readily accessible, simple, low-burden, PRO measure. The use of standardized and widely accepted measures (like the EORTC QLQ-C30) facilitates implementation with minimal additional training for healthcare providers [22]. Standardized PROs can enhance consistency and comparability across different clinical settings. Patients and clinicians should benefit from improved understanding of their prognosis and be better equipped to make decisions regarding their future treatment and care [23]. Model M2 could also be used as a stratification factor in future randomized trials.

Our analyses demonstrated that pain ratings focused on abdominal pain and/or pain related to eating (as captured by the QLQ-STO22, Model M3) offered greater prognostic value than general pain ratings from the QLQ-C30 (Model M2). However, the overall performance of Model M3 was not significantly better than that of Model M2. Additionally, the benefit of using the pain score from the QLQ-C30 over the abdominal pain score of the QLQ-STO22 is reduced responder- and administrator-burden, and easier, streamlined administration of a single, readily accessible instrument (QLQ-C30). These findings warrant further investigation in additional datasets.

Our study has important strengths. We used Cox proportional hazards models with the LASSO method for variable selection. This approach strengthens the model’s reliability, reduces the risk of overfitting, and identifies independent prognostic factors more effectively than previous methods [24, 25]. Variable selection involved both univariable screening and multivariable modeling to account for potential confounders. INTEGRATE IIa and INTEGRATE included participants from Asia, Australia, and Canada resulting in a more diverse study population than studies performed in only one region. We used validated translations of the EORTC PROs in non-English speaking participants.

This study also has limitations. Firstly, the models were built and validated with data from clinical trial populations, which may not be representative of the broader, more heterogeneous population treated outside of clinical trials. [26] Trial participants often differ in terms of demographics, comorbidities, and disease severity, which could limit the model’s generalizability to routine clinical practice. Future studies should test the model’s external validity by applying it in routine clinical settings. Our sample sizes were moderate, and further validation in larger, independent data sets is also warranted [27]. While the model showed good predictive accuracy within our 2 study cohorts, its performance might differ in a broader or more diverse population. All participants were receiving second or subsequent lines of treatment and had an ECOG performance status of 0–1. The applicability of our model to patients with ECOG performance status worse than 1 or having first-line treatment remains as open questions for further research.

We did not perform a decision curve analysis (DCA) and net reclassification improvement (NRI) in this study; alternative methods for assessing model performance and clinical utility in terms of reclassification. These approaches provide additional insights if the proposed application of a model is to categorize participants to directly influence decision making. Future studies of models designed to influence decision making should incorporate DCA and NRI.

Future research should explore the applicability of our model in routine clinical settings to further validate its utility and impact. A web-based tool would facilitate its use and allow real-time validation in clinical practice settings [28]. Future research could assess the integration of this model in an Electronic Health Record to enhance data collection, accessibility, and data-driven decision-making [29]. Training programs for clinicians could facilitate its adoption and implementation with the aim of improving doctor–patient communication and care [30].

In conclusion, we developed and validated a nomogram to predict the probability of survival at 6 months in AGOC being treated with second or subsequent line anti-cancer treatments. By incorporating clinicopathological and patient-reported variables that are simple to collect, and often collected routinely, the model enhanced prognostic accuracy and supports a more personalized approach. Implementation of this nomogram in clinical practice could improve patient care and decision-making by contributing to more frequent and accurate discussions of prognosis, and better stratification in clinical trials.

*GOJ* gastro-esophageal junction, *NLR* neutrophil to lymphocyte ratio, *LDH* lactate dehydrogenase, *CA* carbohydrate antigen.

## Data Availability

The datasets generated during and/or analyzed during the current study are available from the corresponding author on reasonable request.

## Appendix 1

See Tables 4 and 5

**Table 4:** Baseline clinicopathological prognostic factors

Variable	Continuous	Categorical	Range	Coding for analysis
Performance status (ECOG)		Yes	0—5	0 = 0 1 = 1 2 = other if any
Number of prior lines of therapy		Yes	1–2 vs 3 +	0 = 1–2 1 = 3 – 3 +
Neutrophil: lymphocyte ratio	Yes		Disease and contest specific could differ in trial. Might need to be normalized and transformed	
Albumin	Yes		Might need to be normalized and transformed	
BMI	Yes			
Primary disease site		Yes	GOJ vs stomach	0 = Gastro-esophageal junction 1 = Stomach
Region		Yes	Asian vs rest of world	0 = Asia 1 = rest of world
Extent of cancer *(number of metastatic sites)	yes			0 = 1–2 1 = 3–4 2 = 5 + (could redistribute after analysis)
Tumor location (liver)		Yes	Liver vs other	0 = liver 1 = other
Tumor location (Peritoneal/other)		Yes	Peritoneal vs other	0 = peritoneal 1 = other
Baseline tumor markers 19.9 and LDH	Yes		Normal range 0–37 U/ml	
Gastrectomy/ primary resection		Yes	Yes/no	0 = yes 1 = no
Sex		Yes	Male/female	0 = male 1 = female
Age	Yes			

*Extent of cancer was taken from target lesion that might not represent true extent

**Table 5:** Overview of baseline prognostic factors, detailing both continuous and categorical variables, their respective ranges, and coding schemes for analysis. Baseline patient reported outcomes (PROs) tested for possible prognostic significance

Variable	PROM and item number	Continuous range
Appetite loss	QLQ-C30 item 13	Transformed scale score 0–100 (higher = worse)
Appetite loss	STO22 item 34	Transformed scale score 0–100 (higher = worse)
Pain	QLQ-C30 item 9,19	Transformed scale score 0–100 (higher = worse)
Pain	STO22 item 35, 36	Transformed scale score 0–100 (higher = worse)
Fatigue	QLQ-C30 item 10,12,18	Transformed scale score 0–100 (higher = worse)
Constipation	QLQ-C30 item 16	Transformed scale score 0–100 (higher = worse)
Clinicians’ survival estimates		In weeks

EORTC QLQ-C 30 and STO22 scoring range

1 = not at all

2 = a little

3 = quite a bit

4 = very much.

## EORTC scoring

All of the scales and single-item measures range in score from 0 to 100. A high scale score represents a higher response level. Thus, a high score for a functional scale represents a high / healthy level of functioning, a high score for the global health status/QoL represents a high QoL, but a high score for a symptom scale / item represents a high level of symptomatology / problems. Symptom scales / items and Global health status/QoL: Score = {(RS − 1) range} Å ~ 100.

## References

[1] Sung H, Ferlay J, Siegel RL, et al. Global cancer statistics 2020: GLOBOCAN estimates of incidence and mortality worldwide for 36 cancers in 185 countries. CA Cancer J Clin. 2021;71:209–49. 33538338 10.3322/caac.21660

[2] Glare P, Virik K, Jones M, et al. A systematic review of physicians’ survival predictions in terminally ill cancer patients. BMJ. 2003;327:195–8. 12881260 10.1136/bmj.327.7408.195 PMC166124

[3] Epstein AS, Prigerson HG, O’Reilly EM, Maciejewski PK. Discussions of life expectancy and changes in illness understanding in patients with advanced cancer. J Clin Oncol. 2016;34:2398–403. 27217454 10.1200/JCO.2015.63.6696 PMC4981977

[4] Narita Y, Kadowaki S, Oze I, et al. Establishment and validation of prognostic nomograms in first-line metastatic gastric cancer patients. J Gastrointest Oncol. 2018;9:52–63. 29564171 10.21037/jgo.2017.11.08 PMC5848026

[5] Lee J, Lim T, Uhm JE, et al. Prognostic model to predict survival following first-line chemotherapy in patients with metastatic gastric adenocarcinoma. Ann Oncol. 2007;18:886–91. 17298958 10.1093/annonc/mdl501

[6] Hu Z-D, Huang Y-L, Qin B-D, et al. Prognostic value of neutrophil to lymphocyte ratio for gastric cancer. Ann Transl Med. 2015;3:50–50. 25861605 10.3978/j.issn.2305-5839.2015.03.26 PMC4381463

[7] Zhou D, Wu Y, Zhu Y, et al. The prognostic value of neutrophil-to-lymphocyte ratio and monocyte-to-lymphocyte ratio in metastatic gastric cancer treated with systemic chemotherapy. J Cancer. 2020;11:4205–12. 32368303 10.7150/jca.39575 PMC7196266

[8] Gotay CC, Kawamoto CT, Bottomley A, Efficace F. The prognostic significance of patient-reported outcomes in cancer clinical trials. J Clin Oncol. 2008;26:1355–63. 18227528 10.1200/JCO.2007.13.3439

[9] Mierzynska J, Piccinin C, Pe M, et al. Prognostic value of patient-reported outcomes from international randomised clinical trials on cancer: a systematic review. Lancet Oncol. 2019;20:e685–98. 31797795 10.1016/S1470-2045(19)30656-4

[10] Park SH, Cho MS, Kim YS, et al. Self-reported health-related quality of life predicts survival for patients with advanced gastric cancer treated with first-line chemotherapy. Qual Life Res. 2008;17:207–14. 18224458 10.1007/s11136-008-9307-8

[11] Naher SK, Mercieca-Bebber R, Siu D, et al. Prognostic value of patient reported outcomes in advanced gastro-oesophageal cancer: a systematic review. Intern Med J. 2023;53:1946–55. 37605848 10.1111/imj.16209

[12] Chau I, Norman AR, Cunningham D, et al. Multivariate prognostic factor analysis in locally advanced and metastatic esophago-gastric cancer–pooled analysis from three multicenter, randomized, controlled trials using individual patient data. J Clin Oncol. 2004;22:2395–403. 15197201 10.1200/JCO.2004.08.154

[13] Spencer KL, Absolom KL, Allsop MJ, et al. Fixing the leaky pipe: how to improve the uptake of patient-reported outcomes-based prognostic and predictive models in cancer clinical practice. JCO Clin Cancer Inform. 2023;7:e2300070. 37976441 10.1200/CCI.23.00070 PMC10681558

[14] Pavlakis N, Sjoquist KM, Martin AJ, et al. Regorafenib for the treatment of advanced gastric cancer (INTEGRATE): a multinational placebo-controlled phase II trial. J Clin Oncol. 2016;34:2728–35. 27325864 10.1200/JCO.2015.65.1901 PMC5019744

[15] Pavlakis N, Shitara K, Sjoquist K, et al. Integrate IIa phase III study: regorafenib for refractory advanced gastric cancer. J Clin Oncol. 2025;43:453–63. 39365958 10.1200/JCO.24.00055

[16] SAS Institute Inc. 2023. SAS/STAT® 15.3 User’s Guide. Cary N, Inc. SI: SAS Institute Inc. 2023. SAS/STAT® 15.3 User’s Guide. Cary NC: SAS Institute Inc., (ed 9.4), 2023, pp SAS

[17] R Core Team (2021). R A language and environment for statistical computing. R Foundation for Statistical Computing V, Austria

[18] Martin AJ, Gibbs E, Sjoquist K, et al. Health-related quality of life associated with regorafenib treatment in refractory advanced gastric adenocarcinoma. Gastric Cancer. 2018;21:473–80. 28815316 10.1007/s10120-017-0754-1

[19] Balachandran VP, Gonen M, Smith JJ, DeMatteo RP. Nomograms in oncology: more than meets the eye. Lancet Oncol. 2015;16:e173–80. 25846097 10.1016/S1470-2045(14)71116-7 PMC4465353

[20] Steyerberg EW, Vickers AJ, Cook NR, et al. Assessing the performance of prediction models: a framework for traditional and novel measures. Epidemiology. 2010;21:128–38. 20010215 10.1097/EDE.0b013e3181c30fb2 PMC3575184

[21] Crowson CS, Atkinson EJ, Therneau TM. Assessing calibration of prognostic risk scores. Stat Methods Med Res. 2016;25:1692–706. 23907781 10.1177/0962280213497434 PMC3933449

[22] Fayers P, Bottomley A. Quality of life research within the EORTC—the EORTC QLQ-C30. Eur J Cancer. 2002;38:125–33. 10.1016/s0959-8049(01)00448-8 11858978

[23] Vogenberg FR. Predictive and prognostic models: implications for healthcare decision-making in a modern recession. Am Health Drug Benefits. 2009;2:218–22. 25126292 PMC4106488

[24] Tibshirani R. The lasso method for variable selection in the Cox model. Stat Med. 1997;16:385–95. 9044528 10.1002/(sici)1097-0258(19970228)16:4<385::aid-sim380>3.0.co;2-3

[25] Xu Y, Wang X, Huang Y, et al. A LASSO-based survival prediction model for patients with synchronous colorectal carcinomas based on SEER. Transl Cancer Res. 2022;11:2795–809. 36093555 10.21037/tcr-20-1860 PMC9459507

[26] Van Spall HG, Toren A, Kiss A, Fowler RA. Eligibility criteria of randomized controlled trials published in high-impact general medical journals: a systematic sampling review. JAMA. 2007;297:1233–40. 17374817 10.1001/jama.297.11.1233

[27] Dhiman P, Ma J, Qi C, et al. Sample size requirements are not being considered in studies developing prediction models for binary outcomes: a systematic review. BMC Med Res Methodol. 2023;23:188. 37598153 10.1186/s12874-023-02008-1 PMC10439652

[28] Sutton RT, Pincock D, Baumgart DC, et al. An overview of clinical decision support systems: benefits, risks, and strategies for success. NPJ Digit Med. 2020;3:17. 32047862 10.1038/s41746-020-0221-y PMC7005290

[29] Menachemi N, Collum TH. Benefits and drawbacks of electronic health record systems. Risk Manag Healthc Policy. 2011;4:47–55. 22312227 10.2147/RMHP.S12985 PMC3270933

[30] van der Velden NCA, Meijers MC, Han PKJ, et al. The effect of prognostic communication on patient outcomes in palliative cancer care: a systematic review. Curr Treat Options Oncol. 2020;21:40. 32328821 10.1007/s11864-020-00742-y PMC7181418

